# Temporal shifts in the marine feeding of individual Atlantic salmon inferred from scale isotope ratios

**DOI:** 10.1002/ece3.10656

**Published:** 2023-10-31

**Authors:** Michael Power, Eva B. Thorstad, Torbjørn Forseth, Peder Fiske

**Affiliations:** ^1^ Department of Biology University of Waterloo Waterloo Ontario Canada; ^2^ Aquatic Ecology Department Norwegian Institute for Nature Research (NINA) Trondheim Norway

**Keywords:** diet, energetics, feeding, growth, marine migration, *Salmo salar*

## Abstract

Given the limited information on prey use during the marine residency period for Atlantic salmon, scales were collected from salmon at return to the River Namsen (Norway) for spawning after 1 year at sea, and scale material from the first and second summer marine feeding periods was analysed using stable isotope methods to understand dynamics of their trophic ecology. As the salmon increased in size from the first to second summer, they reduced their feeding niche and specialised more (narrowed the δ^13^C range) and increased their dependency on higher tropic level (δ^15^N) prey, likely fish. Changes in δ^13^C indicated a consistent pattern of movement towards the north and west between summer feeding periods. Hence, salmon during their first year at sea may have a migration route roughly resembling that of previous spawners, as inferred from earlier tagging studies. Feeding conditions and nutrient composition during the last summer at sea, i.e. in the months before returning to the river for spawning, impacted final body size and within‐season timing of return. Fish undergoing the largest trophic niche shift (δ^13^C and δ^15^N combined) between summer feeding periods, returned earliest. The earliest returning fish had the fastest specific growth rates at sea. Hence, salmon encountering abundant high‐quality fish food during the marine migration, particularly during the last months, may reach a size and energetic state whereby it is better to return early to a safer environment in freshwater than risk being eaten by a big predator at sea. Both trophic status (δ^15^N), resource use (δ^13^C) and growth rates were significantly correlated between feeding periods. Nutrient composition during the first summer at sea did not impact the fish body length after the following winter, but growth conditions during the first summer evidenced carry‐over effects from the first to the second summer of feeding.

## Graphical abstract

Scale material from the first and second summer marine feeding periods of Atlantic salmon was analysed using stable isotope methods to understand dynamics of their trophic ecology. As fish increased in body size from the first to second summer, they reduced their feeding niche and specialised more (narrowed the δ^13^C range) and increased their dependency on higher tropic level (δ^15^N) prey, likely fish. Feeding conditions and nutrient composition during the last summer at sea in the months before returning to the river for spawning, impacted final body size and within‐season timing of return.

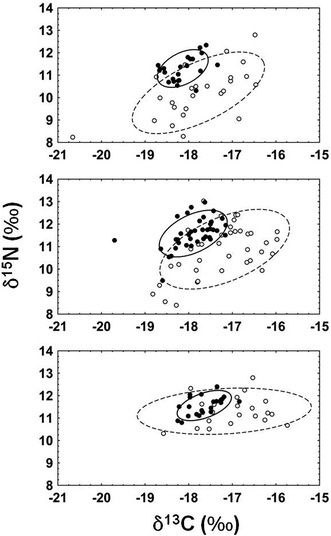

## 1 INTRODUCTION

Atlantic salmon (*Salmo salar*) is a migratory species, accessing marine environments for feeding and growth where conditions allow. Within and among populations, Atlantic salmon display a wide diversity of life‐history strategies, varying in freshwater habitat use (Klemetsen et al., 2003), size‐ and age‐at‐smoltification (Power, 1981), growth (Hutchings & Jones, 1998), length of freshwater residency (Fleming, 1996), age‐at‐maturity (Meerburg, 1986; Power, 1986) and ocean feeding (Jacobsen & Hansen, 2001; Rikardsen & Dempson, 2011). In salmonids, such diversity may represent an evolutionary‐based bet‐hedging strategy, which results in improved population resilience achieved by spatial and/or temporal risk spreading that facilitates continued survival in highly variable and stochastic aquatic environments (Fleming et al., 2014). For example, studies of sockeye salmon (*Oncorhynchus nerka*) have demonstrated that life‐history diversity can increase production and buffer long‐term population fluctuations (Greene et al., 2010). Most analyses for Atlantic salmon consider only variation in life‐history strategies related to migration, reproductive timing and habitat use (e.g. Erkinaro et al., 2019; Persson et al., 2023), and diversity in genetic characteristics (e.g. Fleming et al., 2000; Vähä et al., 2007). Diet, however, can also play a critical role, with dietary plasticity being linked to the ability of Atlantic salmon to survive and prosper in varying environments (Kelly et al., 2019; Renkawitz & Sheehan, 2011).

While researchers have become increasingly aware of the significance of differences in feeding among individuals, most dietary studies of Atlantic salmon have addressed only differences among populations and life‐history groupings, or variation over time. For example, trophic differences exist among populations in closely located rivers (e.g. Sinnatamby et al., 2009). Significant differences in the stable‐isotope values between spawning strategies (i.e. consecutive repeat and alternate spawners) and among rivers have been reported for Atlantic salmon from New Brunswick (Kelly et al., 2019) and Newfoundland (Bøe et al., 2020), which are consistent with differential migration and divergent feeding locations. Differences in diets, in turn, are believed to have possible carry‐over effects on fitness‐related traits such as body condition, parental quality and survival (Harrison et al., 2011). Identification of dietary patterns and their links to survival and growth (Friedland et al., 2009; Utne et al., 2022) are likely to be of increasing importance given the large‐scale changes that have occurred in the ocean food webs (Beaugrand & Reid, 2003) on which Atlantic salmon rely (Beaugrand & Reid, 2012; Dempson et al., 2010) and the importance of food webs for determining fish condition and survival (Frank et al., 2007; Todd et al., 2008; Vollset et al., 2022).

Atlantic salmon are opportunistic feeders that use a wide variety of prey (Renkawitz et al., 2015; Rikardsen & Dempson, 2011; Utne et al., 2021), with prey use varying depending on the spatial and temporal nature of feeding and individual body size (Haugland et al., 2006; Hellenbrecht et al., 2023). Although as a group, Atlantic salmon will eat a wide variety of prey, detailed studies of stomach contents at the postsmolt and adult stages often report only a few different prey items in the stomachs of each individual (Andreassen et al., 2001; Jacobsen & Hansen, 2000), suggesting a degree of selectivity exists among individuals. Such reports are consistent with increasing evidence of intraspecific differences in resource use that can hold significant implications for fitness (Bolnick et al., 2003) and affect both population and community dynamics (Araújo et al., 2011; Dall et al., 2012). Failure to appreciate the degree of intraspecific variation in diet or feeding strategies may result in failure to appreciate the ability of a species to survive in variable or changing environments (Bolnick et al., 2003) or to fully understand niche breadth and overlap (Violle et al., 2012). In Atlantic salmon, differences in feeding have been linked to differences in migration timing (Metcalfe & Thorpe, 1992) and population‐level differences in marine migratory behaviour between northern and southern European salmon populations (Rikardsen & Dempson, 2011). Furthermore, the increasing use of satellite and archival tags has documented variation among individuals from a single population suggestive of the importance of individual experience and encountered ecological conditions for determining growth and survival (Rikardsen et al., 2021; Strøm et al., 2017, 2018).

While electronic tagging methods can be used to provide detailed individual data and describe probable differences in foraging activity related to resource use (e.g. deep versus shallow diving behaviour), studies using these methods are often limited by small sample sizes (Espinasse et al., 2019) and cannot address issues related to resource use and diet directly. Studies of stomach contents can provide a snapshot of the diet but are restricted to the periods and areas of capture (Utne et al., 2021). One means of detecting differences in diet within and among groups of fish over space and time is by analysing the stable isotopes of their scales (e.g. Kelly et al., 2019; Torniainen et al., 2014). Based on rearing experiments, Atlantic salmon stable isotopes vary in composition with variations in nutritional and maturation status (Grahl‐Nielsen & Glover, 2010). Thus, the isotopic composition of the scale will vary with changes in the food web over time and space. Furthermore, spatial gradients in oceanic δ^13^C are connected to variations in CO_2_ availability (Francois et al., 1993; Rau et al., 1982) and temperature (Sackett et al., 1965), whereas gradients in oceanic δ^15^N depend on nutrient concentrations and sea surface temperatures (Hetherington et al., 2017). Predictable changes across the gradients predispose stable isotopes for use in movement and location studies (e.g. Hobson, 1999). Thus, stable isotopes based on scale analyses are likely to be particularly useful for aquatic species such as Atlantic salmon that may be easily intercepted and nonlethally sampled during the migration process, or for which long‐term sample archives exist (e.g. Espinasse et al., 2019; Kelly et al., 2019; Sinnatamby et al., 2009; Torniainen et al., 2014).

Here, we use stable isotope (δ^13^C, δ^15^N) data obtained from scales sampled from wild Atlantic salmon at return to the river after 1 year at sea to describe the relationships between body size, return timing and trophic status when feeding in the marine environment. Specifically, we predict that: (1) larger smolts will feed at higher trophic levels at sea because of the advantages conferred by size; (2) as individuals increase in size during the marine migration they will specialise more and feed at higher tropic levels such that: (a) δ^15^N will increase with size within and between summer feeding periods, and (b) trophic niche size will decrease between the first and second summer feeding periods, because of a reduced dietary diversity and increasing use of fish prey with increasing body size; (3) carry‐over effects between the first and second feeding season will be evident in a strong positive correlation between the individual trophic status of the first and second summers and have consequences for growth; and (4) differences in return‐timing will be reflected in differences in trophic ecology, growth and condition, with earlier returning fish having the most specialised trophic niche and the highest growth rate and condition.

## 2 METHODS

Scales from Atlantic salmon were obtained from fish returning to the River Namsen in Central Norway (river mouth 64.457° N, 11.506° E) in 2017. The Atlantic salmon were captured in bag nets in Namsfjorden, immediately prior to river entry, or in the fish ladder at the Nedre Fiskumfoss waterfall located in the river 70 km from the sea. Only one‐sea‐winter wild salmon were included in the study, that is salmon that had stayed 1 year at sea before returning to the river. All samples consisted of dead fish caught and killed by licenced fishers and were not regarded under Norwegian legislation as research animals requiring a use and care permit from the Norwegian Animal Research Authority. All obtained samples were subsequently classified as wild or escaped from aquaculture farms based on growth patterns on their scales (Fiske et al., 2005) and farmed fish were excluded from all analyses.

Scales (*n* = 8–10 from each fish) were cleaned to remove any surface mucous residue by soaking in de‐ionised water and gently rubbing between the forefinger and thumb. For analysis, two different sections were removed from each scale by cutting with a scalpel, with each section corresponding to a marine growth zone associated with summer feeding at sea. The location of the cuts was determined based on scale circuli spacing following the protocols described in Power (1987). Unique scalpel blades were used for each fish. Scale material obtained from each growth zone was shredded using sterile laboratory scissors to obtain the weight of material required for stable isotope analysis (0.29–0.31 mg). Scales were not acidified prior to use in analyses because the inorganic fraction of the scale has been demonstrated to have no significant effect on the obtained stable isotope measures for marine salmon (MacKenzie et al., 2011, 2012; Sinnatamby et al., 2009). Although Atlantic salmon scales contain lipids (Grahl‐Nielsen & Glover, 2010), the overall amounts are typically considered too small to affect overall isotopic composition (Espinasse et al., 2019). For aquatic animals, lipid correction (e.g. Kiljunen et al., 2006) is not completed when sample C:N ratios are <3.5 (Post et al., 2007), as was the case for all samples in this study.

Scale sample masses for analyses were determined using a Mettler–Toledo model XP2U micro‐analytical balance (Mettler–Toledo GmbH) and were used in the simultaneous analysis of stable carbon (δ^13^C) and nitrogen (δ^15^N) isotopes (SIA) on a Delta Plus Continuous Flow Stable Isotope Ratio Mass Spectrometer (Thermo Finnigan) coupled to a 4010 Elemental Analyser (CNSO 4010; Costech Analytical Technologies Inc.). Duplicates were run every 10th sample for quality assurance purposes. Internal laboratory standards cross‐calibrated against the International Atomic Energy Agency standards for carbon (CH3, CH6) and nitrogen (N1, N2) were inserted at the beginning, middle, and end of each sample run to ensure reportable measurement accuracy determined as ±0.2‰ and ± 0.3‰, respectively, for δ^13^C and δ^15^N. All results are quoted in conventional delta notation (δ) and expressed relative to Vienna Peedee Belemnite limestone for δ^13^C (Craig, 1957) and atmospheric nitrogen for δ^15^N (Mariotti, 1983).

As scales grow in three dimensions via the successive accumulation of collagen layers by underplating (Ikoma et al., 2003; Zylberberg & Nicolas, 1982), they increase in thickness posteriorly towards the focus, because the older parts of the scale are overlaid by new collagen (Hutchinson & Trueman, 2006). The process of temporal overlaying of collagen biases the stable isotope signatures of material used to represent earlier feeding periods, such that the stable isotope values obtained from the first summer feeding period must be corrected to account for collagen overlay from the second summer feeding period (Dixon et al., 2015). We corrected the first summer feeding stable isotope values to account for collagen overlaying by applying the corrective mathematical model developed by Dixon et al. (2015) after checking for isometric growth.

Fish age and size‐at‐age were determined from image analysis (Image Pro 6.3©software; Media Cybernetics) of scales collected from the dorsal area of the fish close to the lateral line between the dorsal and adipose fins. The region is where scales first form on salmonid fishes (Elson, 1939), with sampling of scales from this region allowing the most complete view of the growth history of an individual fish. For each fish, a single, easily readable scale was selected for circuli spacing analysis following methods described by Jensen et al. (2012). Measures were made of spacing from the centre of the scale to the circulus representing smoltification and sea entry, to the circulus representing the end of the first winter at sea, and to the outer edge of the scale. The fish body length at smoltification and at the end of the first winter at sea were then estimated by back‐calculation of fish length using the linear relationship between fish length and scale radius as described in Hanson et al. (2019). Back‐calculated lengths were used to estimate specific growth rate as the difference between the logarithms of length in adjacent periods divided by the time interval in days and multiplied by 100 (Lugert et al., 2016). For the first feeding season, specific growth rate was computed using length at smoltification and length at the end of the first winter. For the second feeding season, specific growth rate was computed using length at the end of the first winter and length at capture. For the second summer at sea, the onset of growth was assumed to start at a fixed date set equal to the midpoint (Day 60) of the mid‐February and late March range suggested for annulus formation in marine overwintering salmon (Carlson et al., 2021).

Data obtained from stable isotope analyses were used to characterise changes in temporal feeding patterns occurring between the first and second summer at sea and between return timing dates (i.e. July, August and September) with data describing the δ^13^C and δ^15^N feeding traits (sensu Guillerme et al., 2020) of individual fish. The significance of correlations between back‐calculated length‐at‐smoltification, length at the end of the first sea winter and length‐at‐capture and the relevant period δ^15^N were assessed with simple linear regression (Zar, 2010). The strength of between‐period correlation in the isotope data indicative of carry‐over effects was similarly assessed with linear regression. Multiple comparisons of means were completed using one‐way ANOVA (Zar, 2010) where necessary. All statistical testing described above was completed using Statistica release 8 (StatSoft Inc.).

For Atlantic salmon captured in July and August, the catch date was considered to accurately represent return timing to the river, because earlier radio tagging studies have shown that Atlantic salmon enter the river about 1 day after they are captured and released in the estuary, with migration speeds estimated from a logging station located 11 km from the river mouth indicating average ± SD speeds of 20.6 ± 15.3 km/day (Thorstad et al., 1998, own unpublished data). For the fish captured in the river in September, only a few days may have been spent reaching the upstream Nedre Fiskumfoss fish ladder based on the earlier Thorstad et al. (1998) study, but we do not know if fish were further delayed below the fish ladder where they were captured. Upstream migration to the fish ladder is known to be fast (i.e. a few days) owing to the lack of migration barriers (Thorstad et al., 1998). Thus, for the group of fish sampled in September, we suppose they entered the river later than those captured in July and August, but we cannot rule out the possibility that some individuals in the group may have had a river entry timing overlapping with the August group.

Stable isotope data were used to define the spread of individuals within isotope space (i.e. feeding niche size), the density of individuals within a given niche space and the position of the defined niche spaces relative to one another. The spread of individuals in isotope space was measured using the sum of the variances (Foote, 1992), the sum of the ranges (Foote, 1992) and the standard ellipse area corrected for small sample sizes, SEA_C_ (Jackson et al., 2011). The use of multiple measures has been argued to provide more nuanced and detailed characterizations of differences in trait space (i.e. feeding niche) occupancy (e.g. Guillerme et al., 2020).

Measures of feeding niche size were supplemented with measures of niche density and position. Niche density was measured by average nearest neighbour distance (e.g. Foote, 1992; Layman et al., 2007) and with the series of metrics proposed by Layman et al. (2007) to quantify the trophic ecology of individuals based on return date. Finally, circular statistical methods (Batschelet, 1981) were used to assess the significance of differences in temporal niche shifts (e.g. Schmidt et al., 2007) using the Watson–Williams *F*‐test for differences in the mean angles to describe the shift in isotopic space between feeding season (Watson & Williams, 1956) as implemented in PAST version 4.08 (Hammer et al., 2001).

## 3 RESULTS

Scale samples were obtained from 92 Atlantic salmon captured in the months of July, August and September. Of those, 86 individuals were retained for analyses. The remaining six individuals were identified as escaped farmed salmon. Summary biological characteristics by month of return and stable isotope values of the fish are reported in Table 1.

**TABLE 1:** Mean ± standard deviation of the biological characteristics of returning River Namsen Atlantic salmon at capture: length, mass, length at smoltification and isotope measurements (δ^13^C, δ^15^N) by month of capture obtained from scale collagen laid down during the first and second summers of feeding at sea, the former corrected for the effect of overgrowth of second summer material.

	Length (cm)	Mass (g)	Smolt (cm)	First summer		Second summer	
				δ^13^C	δ^15^N	δ^13^C	δ^15^N
July *n* = 25	47.7 ± 2.7	787 ± 109	13.0 ± 2.0	−17.8 ± 0.9	10.2 ± 1.1	−18.1 ± 0.4	11.3 ± 0.5
August *n* = 39	50.2 ± 3.4	985 ± 261	13.7 ± 1.9	−17.2 ± 0.9	10.9 ± 1.1	−17.9 ± 0.5	11.6 ± 0.7
September *n* = 22	51.8 ± 3.8	1077 ± 297	13.3 ± 1.9	−17.2 ± 1.2	11.3 ± 0.6	−17.6 ± 0.4	11.6 ± 0.4

**FIGURE 1:**
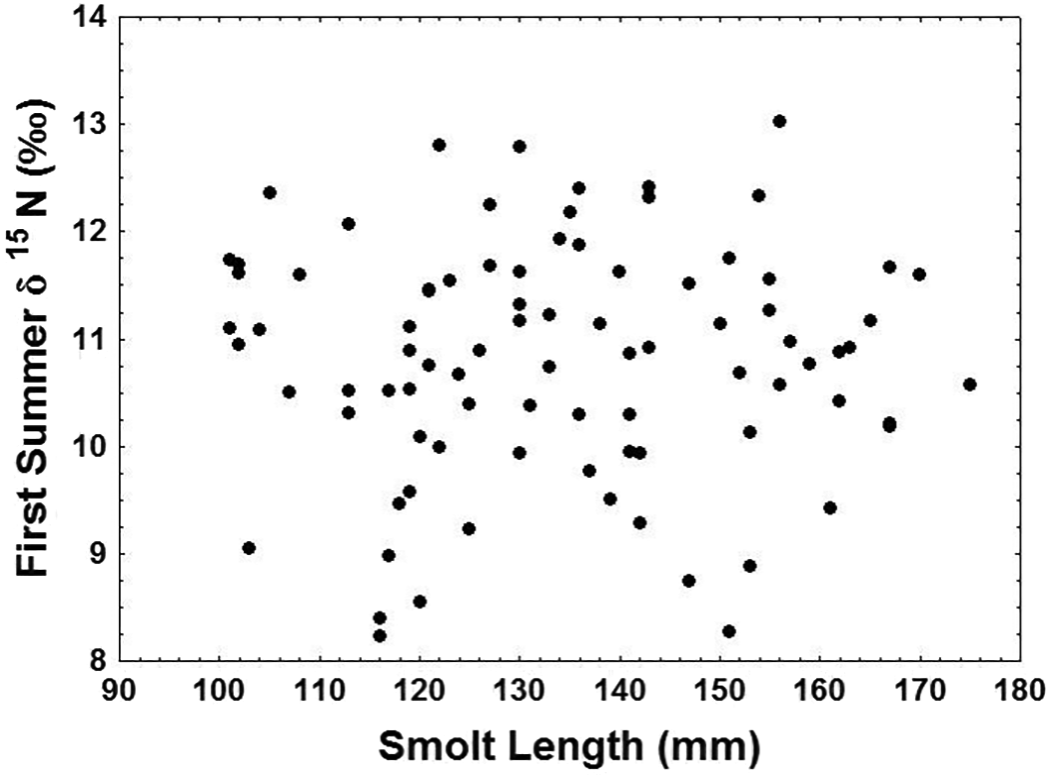
Plot of River Namsen smolt sizes at sea entry versus the trophic (δ^15^N) level attained based on the first summer of marine feeding. Scale δ^15^N values were corrected for underplating following procedures described in Dixon et al. (2015). The relationship between the plotted variables was insignificant (*p* = .88) and explained a low percentage of the variation (<1%).

Back‐calculated body length‐at‐smoltification varied more (CV = 14.4%) than body length at the end of the winter (CV = 6.9%), body length‐at‐capture (CV = 7.2%) and trophic diversity during the first (δ^15^N CV = 10.1%) and second (δ^15^N CV = 5.0%) summer feeding periods. The trophic variation (δ^15^N) among individuals was not explained by smolt length, given the lack of correlation between smolt body length and δ^15^N during the first summer feeding period (*r* ^2^ < .01, *p* = .88, Figure 1). The back‐calculated body length at the end of the winter was not related to mean δ^15^N during the first summer feeding period (*r* ^2^ < .04, *p* = .06). Body length‐at‐capture increased with increasing mean δ^15^N during the second summer feeding period (*r* ^2^ < .20, *p* < .01). Individual changes in mean δ^15^N between feeding periods, however, were not correlated with between‐feeding period changes in body length (*r* ^2^ < .01, *p* = .61).

**FIGURE 2:**
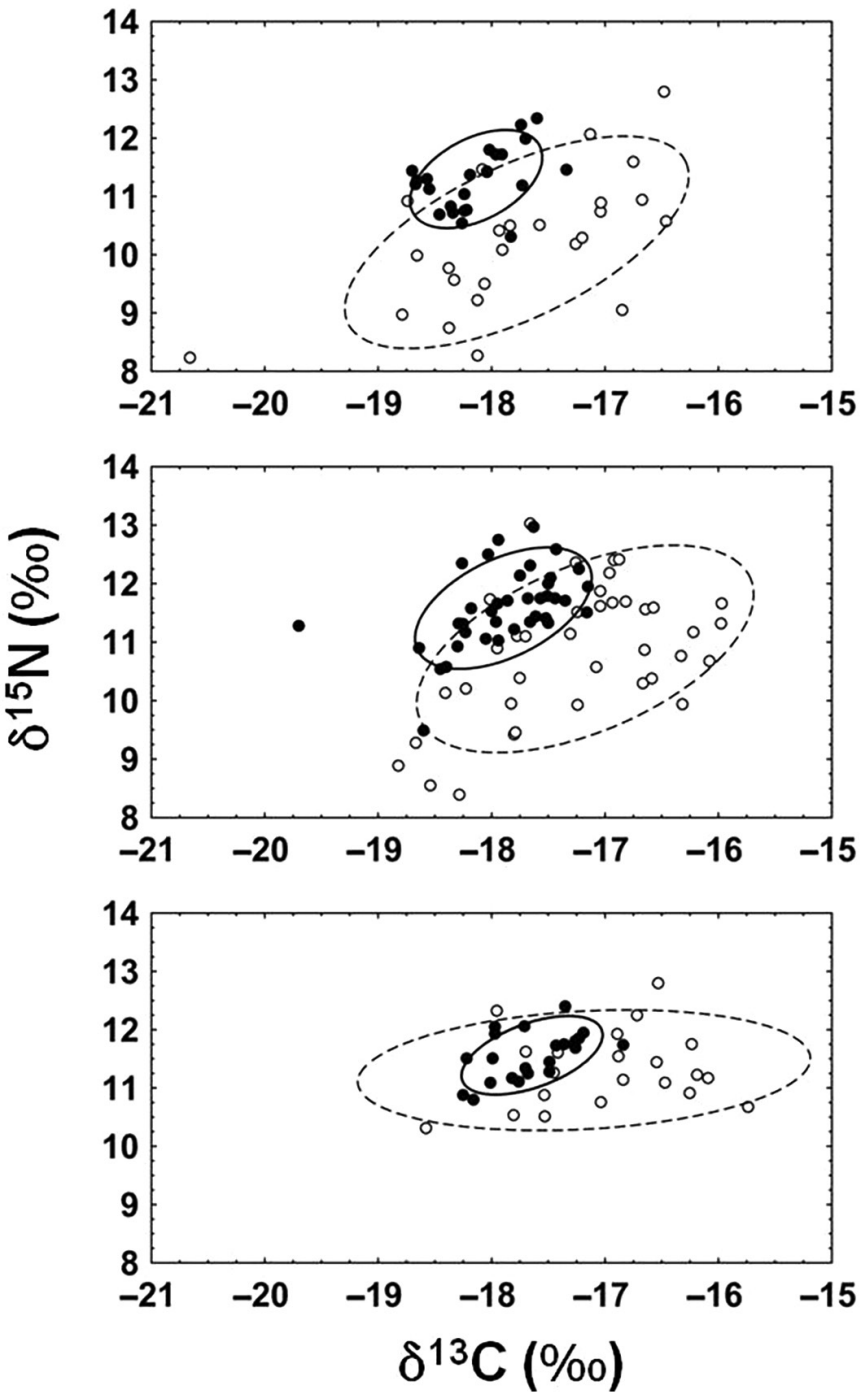
Scale‐based standard ellipses (SEA_C_) for July (top, *n* = 25), August (middle, *n* = 39) and September (bottom, *n* = 22) returning River Namsen Atlantic salmon for the first (○, dashed line) and second (•, solid line) summer feeding periods showing the shrinkage of trophic niche between summer feeding periods.

Trophic niche size as measured by the sum of the variances, the sum of the ranges or SEA_C_ declined from the first summer feeding period to the second summer feeding period (Figure 2) for all fish regardless of which month they returned to the river, with declines varying between 39% and 83% (Table 2) depending on the measure. There were no differences between the return months in the average percentage shrinkage of trophic niche size (*F*_2,6_ = 1.29, *p* = .34). Niche diversification (δ^13^C range), trophic diversity (δ^15^N range) and trophic dissimilarity (MNN) all similarly decreased between feeding seasons, with declines varying between 25% and 76% depending on the measure.

**TABLE 2:** Trophic trait space measures for returning River Namsen Atlantic salmon at capture: SEA_C_, sum of variances, sum of ranges, δ^13^C and δ^15^C range and mean nearest neighbour (MNN).

Measure	First	Second	% Change
July			
SEA_C_	2.717	0.554	−79.6
Sum of variances	1.469	0.635	−56.8
Sum of ranges	94.842	35.041	−62.7
δ^13^C range	4.20	1.36	−67.6
δ^15^N range	4.56	2.03	−55.5
MNN	0.485	0.144	−70.3
August			
SEA_C_	2.920	0.916	−68.6
Sum of variances	1.185	0.709	−39.2
Sum of ranges	151.590	89.131	−41.2
δ^13^C range	4.97	2.55	−48.7
δ^15^N range	4.64	3.48	−25.0
MNN	0.320	0.206	−35.6
September			
SEA_C_	2.518	0.431	−82.9
Sum of variances	0.703	0.190	−73.0
Sum of ranges	44.528	22.987	−48.4
δ^13^C range	5.93	1.41	−76.2
δ^15^N range	2.49	1.60	−35.7
MNN	0.506	0.152	−69.9

*Note*: Also given are percent changes for each measure expressed as a percentage of the first summer feeding season measure.

**FIGURE 3:**
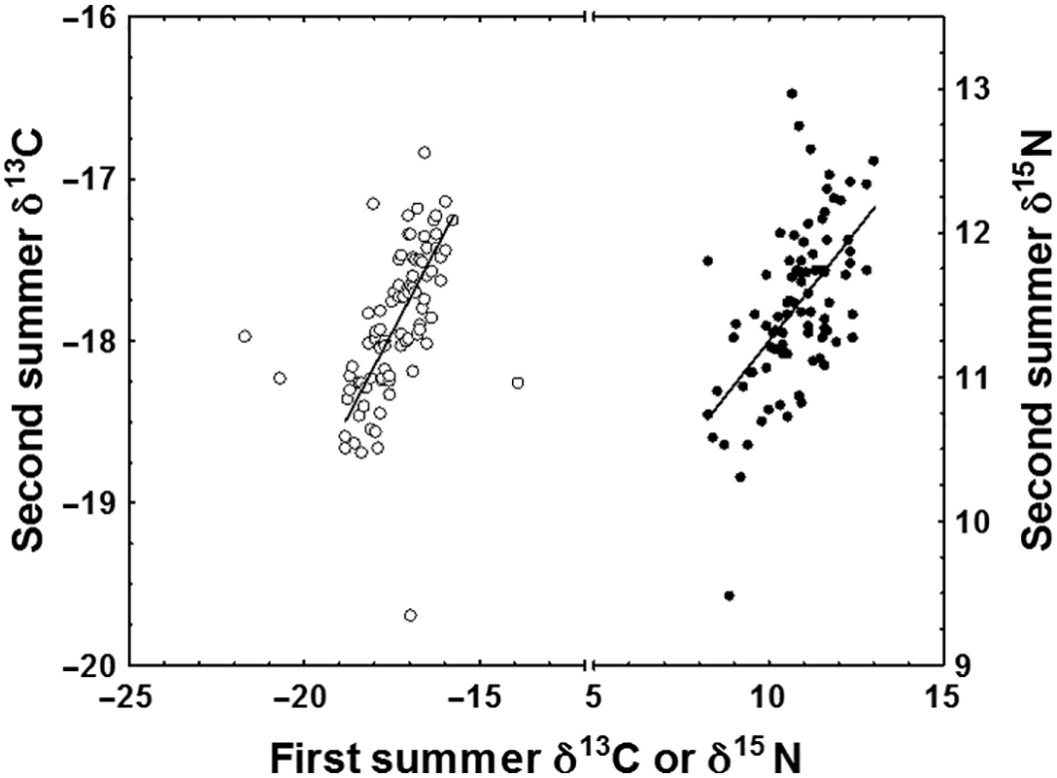
First summer feeding period scale δ^13^C (○) and δ^15^N (•) values for River Namsen Atlantic salmon plotted against like scale values for the second summer feeding period. First summer feeding period values are corrected for the effect of the over‐growth of second summer material. Solid lines plot significant regression lines (regression *p* < .001). For δ^13^C: δ^13^C Second Summer = −11.676 + 0.357 δ^13^C First Summer. For δ^15^N:δ^15^N Second Summer = 8.181 + 0.309 δ^15^N First Summer.

Trophic status during the second summer feeding period was positively correlated with trophic status during the first summer feeding period (δ^15^N: *r* ^2^ = .35, *p* < .01, Figure 3). Similarly, resource use during the second summer feeding period was positively correlated with resource use during the first summer feeding period (δ^13^C: *r* ^2^ = .52, *p* < .01 Figure 3). Changes in δ^13^C and δ^15^N between feeding periods were not significantly correlated (*r* ^2^ = .03, *p* = .09). Specific growth rate during the first and second summer feeding periods, however, were significantly and positively correlated (*r* ^2^ = .92, *p* < .01).

**FIGURE 4:**
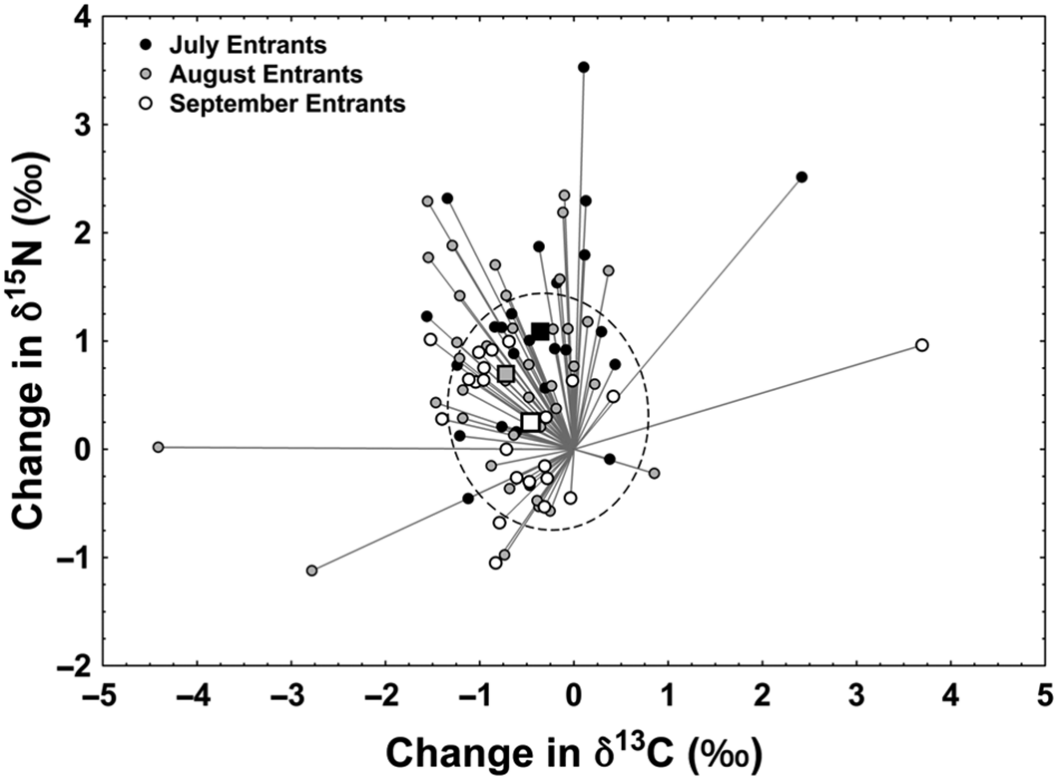
Directional shift vectors for River Namsen returning Atlantic salmon sorted by month of presumed return. Vectors plot the combined direction and magnitude of changes in scale δ^13^C and δ^15^N for individual fish from the first to the second summer feeding periods. Squares plot the mean vector shift for all fish in each month. The plotted ellipse corresponds to the SEA_C_ for the vector endpoints denoted by the small, filled circles.

The mean angular change in isotopic space (i.e. the angle formed by the combined shift in δ^13^C and δ^15^N between summer feeding periods) differed significantly between return months (Watson‐Williams *F*_2,83_ = 5.37, *p* = .01), with fish returning in July showing the largest trophic shift (+1.09‰) and those returning in August showing the largest change in δ^13^C (−0.72‰) (Figure 4). The mean Euclidean shift in isotopic space was largest for the July fish (1.14) and smallest for the September fish (0.52). Earlier (July) returning fish also displayed large reductions in both niche diversification (δ^13^C range) and trophic diversity (δ^15^N range), whereas August and September returning fish showed large reductions in only niche diversification.

Specific growth rates in both the first and second summer feeding periods differed by month of return (first summer: ANOVA *F*_2,83_ = 25.35, *p* < .01; second summer: ANOVA *F*_2,83_ = 26.53, *p* < .01), declining from July (first summer: 0.71; second summer: 0.91) through September (first summer: 0.53; second summer: 0.71). There were no accompanying differences in condition (Fulton's *K*) by month of return (ANOVA *F*_2,83_ = 2.43, *p* = .09).

## 4 DISCUSSION

Based on analysis of their scales, Atlantic salmon experienced significant trophic shifts (changes in either δ^13^C or δ^15^N) between the first and the second summer of marine feeding. Since these were salmon returning to the river for spawning after only 1 year at sea, the second summer stable isotope signature reflected the last months at sea before the fish returned to the river. Observed trophic shifts were not related to smolt length at the time of marine entry. Furthermore, achieved trophic position at river return was not related to body length at the end of the first summer of feeding, but was related to body length by the end of the second summer of feeding. Given the correlation between achieved trophic level and resource use in the first and second summers of feeding, the length‐dependent trophic positioning of individual fish points to carry‐over effects from the first summer of feeding.

Changes in the size of the feeding niche during the marine migration period were evident, with fish in this study significantly reducing their feeding niche as they grew and increasing their dependency on higher tropic level prey, likely fish. Among‐river comparisons of Canadian populations have highlighted the high pelagic reliance of marine feeding salmon (Dixon et al., 2012). Based on the reduction in δ^13^C and δ^15^N ranges, Dixon et al. (2012) suggested that Atlantic salmon undergo a shift from generalist freshwater feeding to more specialised pelagic zone feeding in the marine environment. The significant reductions in trophic niche space noted in Dixon et al. (2012), occurring at the transition from freshwater to marine feeding, appears to be part of an ongoing process of increased feeding specialisation by Atlantic salmon in the marine environment. The increasing specialisation with size is reflective of a more common pattern exhibited by larger marine predators. For example, decreasing trophic‐niche breadths with ontogeny have been observed in fishes >50 cm (Scarf et al., 2020), which is within the size range of the returning adult Atlantic salmon studied here. Increasing specialisation with size may be reflective of ecological pyramids, with the consistent decreases in production observed with increasing tropic level (Trebilco et al., 2013) implying an ever‐decreasing diversity of suitable prey as fish increase their mean trophic level. In a study of the diet of Atlantic salmon during the last part of the ocean migration along the coast of northern Norway, shortly before river entry and spawning, 95% of bulk stomach contents comprised four different marine fish species including: sand eels (*Ammodytes* spp.), capelin (*Mallotus villosus*), herring (*Clupea harengus*) and haddock (*Melanogrammus aeglefinus*) (Aykanat et al., 2020). Aykanat et al. (2020) further noted that Atlantic salmon switched to the so‐called ‘feast and famine’ strategy as a function of ontogeny, with older age groups exhibiting heavier stomach content that came at the expense of running on empty more often.

Contrary to expectations we did not find that larger smolts fed at higher tropic levels than smaller smolts during their first summer at sea, as no relationship between the first feeding season δ^15^N and body length‐at‐smoltification was observed. In fisheries ecology, the contention that larger or faster growing members of a cohort gain a survival advantage is both intuitive and well established (Sogard, 1997), with the influence of size on Atlantic salmon ecology and life‐history strategies being similarly well established. Thus, increasing body size has been associated with increasing selectivity and use of more profitable prey in stream‐dwelling juveniles (Keeley & Grant, 1997). Body size has also been shown to affect postsmolt survival in many populations (Halfyard et al., 2013). In the ocean, size may determine survival, with larger smolts being less exposed to predation (Saloniemi et al., 2004; Simmons et al., 2021) and able to access a wider variety and quantity of prey (Vehanen et al., 1993). Furthermore, in salmonids, there is a consistent pattern of increasing use of larger prey as body size increases (Keeley & Grant, 2001). Prevalent relationships between body size and δ^15^N in marine food webs (Jennings et al., 2008), therefore, suggested a probable positive relationship between δ^15^N and length‐at‐smoltification. The lack of such a relationship in our data may relate to the immediate loss of size‐related feeding advantages when switching to marine habitats where salmonids in general begin to consume fish at smaller size than in freshwater (Keeley & Grant, 2001). Studies of marine feeding in Norwegian postsmolts similarly note the absence of a critical size threshold for possible piscivory, noting that even some of the smallest post‐smolts (10 cm) were found to consume fish as prey (Rikardsen et al., 2004). The ability of both smaller and larger postsmolts to access fish when fish larvae of a suitable size are present would result in the loss of trophic distinction by body size in fish ranging in length from 10 to 18 cm, as fish in this study did. Hence, variation in the abundance of fish larvae available as prey may be a more important influence on the diet of Atlantic salmon postsmolts than their own body size. The supposition is partly supported by a recent study of early post‐smolt diet by Hellenbrecht et al. (2023) who noted post‐smolts focus their feeding on fish larvae when encountering a high abundance of the prey group. Alternatively, results here may simply reflect a selective effect, with those postsmolts that successfully switch to piscivory early regardless of size tending to survive better than those that do not. Nevertheless, the advantages of large body size appeared to become re‐established over time in our study, as evidenced by the positive relationship between δ^15^N and body size during the second summer feeding period.

A lack of correlation between smolt size and trophic level (δ^15^N) may also occur if fish making a quick and early transition to piscivory are those that tend to survive and return. Reliance on scales from successfully returning fish, as in this study, may bias results by effectively removing any apparent influence of body length on trophic function. There is a large spatial and temporal variation in postsmolt diet in the fjords immediately after sea entry, and a piscivorous diet is known to enhance growth and ultimately survival (Hellenbrecht et al., 2023). Postsmolts during the early marine migration seem to focus their feeding on fish larvae when encountering a high abundance of this prey group and have a diverse diet consisting of several different prey groups when fish larvae are absent (Hellenbrecht et al., 2023). For postsmolts in the Norwegian Sea later in the summer and early autumn, reduction of highly energetic fish larvae and amphipoda in the diet is related to reduced stomach fullness and poorer condition (Utne et al., 2021). Thus, the energetic advantage gained by piscivory facilitates faster growth, with faster growth correlated with higher survival as has been noted for migratory Chinook salmon, *Oncorhynchus tshawytscha* (Duffy & Beauchamp, 2011).

Observed mean shifts in δ^13^C (−0.36 to −0.72 L) and δ^15^N (0.25–1.14 L) between the first and second summer at sea point to persistent increases in the mean trophic level at which fish fed and a shift in either resource use, or the geographic location of feeding, or both. Within‐season shifts in resource use have been documented for Atlantic salmon at West Greenland using shifts in δ^13^C, with decreased δ^13^C indicative of greater reliance on offshore resources (Dixon et al., 2019). The timing and trend of shifts in δ^13^C, and use of inshore resources, were interpreted to reflect the ability of Atlantic salmon to locate and exploit areas of high productivity for purposes of feeding (Dixon et al., 2019), that is nearshore habitats dominated by coastal upwelling and terrestrial inputs that yield high primary production and attract large numbers of potential prey fishes (Grønkjær et al., 2019; Lundin & Lindén, 1993).

Atlantic salmon moving either West or North could also explain the negative δ^13^C shifts noted here, which indicates that salmon during their first year at sea may have a migration route roughly resembling that of previous spawners. The marine migration routes of previous spawners have been mapped with satellite tags (Rikardsen et al., 2021; Strøm et al., 2018), but the routes of first‐time migrants remain largely unknown. The δ^13^C changes observed here exceed those observed by Dixon et al. (2019) and suggest more extensive spatial movements. Our data align more with the results of spatially integrated studies of ocean particulate organic carbon in Arctic waters that show δ^13^C values fall consistently between 40° N and 80° N (Goericke & Fry, 1994). Similarly, δ^13^C measures of mean ocean plankton show decreasing values moving north from the Norwegian to Barents seas and west from the Norwegian Sea to coastal eastern Greenland (Magozzi et al., 2017). Lower sea surface temperatures, higher wind speeds and productivity gradients also appear to enhance atmospheric CO_2_ uptake in Arctic marine waters (Takahashi et al., 2002) creating latitudinal gradients in ocean δ^13^C values reflected across multiple trophic levels (de la Vega et al., 2019). Both possibilities are consistent with the reported movements of previous spawners to ocean habitats as far west as Jan Mayen and Iceland and north to Svalbard (Rikardsen et al., 2021; Strøm et al., 2018). Studies of the early marine distribution of Atlantic salmon in the North‐east Atlantic based on genetic assignment techniques report a similar pattern dominated by progressive northward movement of postsmolts from the Norwegian Sea, although the proportion of Norwegian fish in that study was unexpectedly low (Gilbey et al., 2021).

Models of the global distribution of marine nitrogen isotopes suggest geographic gradients for particulate organic matter (POM) δ^15^N and plankton δ^15^N that show limited variation across the North Atlantic from the Norwegian to the Barents seas (Buchanan et al., 2022; McMahon et al., 2013), although uncertainties regarding the processes that can affect δ^15^N make it hard to conclusively interpret regional patterns (Somes et al., 2010). At depth, POM δ^15^N increases and may account for changes as large as 5–10‰ over the depth range 0–1000 m (Mitenbeck et al., 2007). Changes in δ^15^N observed here must, therefore, be interpreted with caution. Although changes related to geographical shifts in feeding, as indicated by changes in δ^13^C are likely to have been small, changes related to feeding at higher trophic levels or increasingly at depth are both possible and consistent with the directional changes in δ^15^N observed here between feeding seasons. Furthermore, pop‐up satellite archival tags monitored postsmolts showed increases in diving depth (>200 m) during winter and spring (December–April) consistent with deeper foraging (Hedger et al., 2017).

The results in this study emphasised the importance of the feeding conditions during the last summer at sea, that is in the months before returning to the river for spawning, because feeding conditions during this period appear to impact the size of the fish at return and the within‐season timing of return. Specifically, the trophic position attained during the last summer at sea was positively correlated with final body size of the fish. Fish undergoing the largest trophic niche shift, that is the combined shift in δ^13^C and δ^15^N between summer feeding periods, returned earliest in the season. The earliest returning fish reduced their feeding niche and increased their dependency on higher tropic level prey, likely fish, to a larger extent than later returning fish. The earliest returning fish also grew the fastest while feeding at sea. Atlantic salmon stop feeding when they enter the river and until spawning, with the reasons for river entry and feeding cessation several months prior to spawning not being clearly understood at present (Jacobsen & Hansen, 2000: Klemetsen et al., 2003). However, the benefit of good feeding and growth conditions at sea comes at the cost of higher predation risks as compared to freshwater, since there are more larger predators capable of eating adult Atlantic salmon in the ocean than in freshwater. Hence, it might be that the Atlantic salmon encountering abundant high‐quality fish food during the marine migration, particularly during the last months, reach a size and energetic state whereby the risk–reward ratio of continued foraging favours early return to safer freshwater environments.

Although trophic position during the first summer at sea did not impact fish body length at the end of the first winter at sea, relationships between trophic position and body size were evident by the date of return to the river. Furthermore, the correlation between achieved trophic level and resource use in the first and second summers of feeding, the length‐dependent trophic positioning of individual fish points to carry‐over effects from the first to the second summer of feeding.

## 5 CONCLUSION

The use of easily recoverable scales from monitoring programmes, or sample archives, for stable isotope analyses can provide insights into the trophic ecology of Atlantic salmon while at sea, with the notable zonation in the scale facilitating the recovery of material that can be used for period‐to‐period comparisons within the life of a single individual. Using scales obtained from salmon returning to the river after 1 year at sea, we were able to demonstrate that among surviving fish, larger length at smoltification did not confer a size advantage allowing fish to feed at a higher trophic level at marine entry. We found that as Atlantic salmon increase in size they tend to specialise more, as reflected in a narrowing of the observed δ^13^C range, and to feed at higher tropic levels. Furthermore, correlations observed in the isotope data between summer feeding periods suggest significant carry‐over effects between feeding periods, with July returning fish appearing to achieve higher trophic status (δ^15^N) earlier and grow more quickly as a consequence. Thus, differences in within‐season return‐timing were reflected in differences in trophic ecology and growth of 1 sea winter Atlantic salmon. The broader use of scale sampling for trophic studies, therefore, may facilitate the acquisition of a greater understanding of both among‐individual and among‐population variation in the fate of marine feeding Atlantic salmon, and when used in combination with archival samples has the potential to provide insights into the effects of ecosystem shifts on Atlantic salmon. Accordingly, we would encourage the use of scale‐based stable isotope studies as a cost‐effective means of studying the otherwise difficult to study marine feeding phase of the life‐history of Atlantic salmon.

## AUTHOR CONTRIBUTIONS

**Michael Power:** Conceptualization (equal); data curation (lead); formal analysis (lead); funding acquisition (equal); investigation (lead); methodology (equal); project administration (equal); visualization (equal); writing – original draft (lead); writing – review and editing (equal). **Eva B. Thorstad:** Conceptualization (equal); funding acquisition (equal); investigation (supporting); project administration (equal); writing – original draft (equal); writing – review and editing (equal). **Torbjørn Forseth:** Conceptualization (equal); investigation (equal); writing – review and editing (equal). **Peder Fiske:** Conceptualization (equal); investigation (equal); writing – review and editing (equal).

## Data Availability

The data that support the findings of this study are openly available in DRYAD at http://doi.org/10.5061/dryad.hhmgqnknv.
